# MRl of Prostate Cancer Antigen Expression for Diagnosis and lmmunotherapy

**DOI:** 10.1371/journal.pone.0038350

**Published:** 2012-06-27

**Authors:** Jing Ren, Fang Wang, Guangquan Wei, Yong Yang, Ying Liu, Mengqi Wei, Yi Huan, Andrew C. Larson, Zhuoli Zhang

**Affiliations:** 1 Department of Radiology, Xijing Hospital, Fourth Military Medical University, Xian, China; 2 Department of Microbiology, Fourth Military Medical University, Xian, China; 3 Department of Radiology, Northwestern University. Chicago, Illinois, United States of America; 4 Robert H. Lurie Comprehensive Cancer Center, Chicago, Illinois, United States of America; Faculté de médecine de Nantes, France

## Abstract

**Background:**

Tumor antigen (TA)–targeted monoclonal antibody (mAb) immunotherapy can be effective for the treatment of a broad range of cancer etiologies; however, these approaches have demonstrated variable clinical efficacy for the treatment of patients with prostate cancer (PCa). An obstacle currently impeding translational progress has been the inability to quantify the mAb dose that reaches the tumor site and binds to the targeted TAs. The coupling of mAb to nanoparticle-based magnetic resonance imaging (MRI) probes should permit *in vivo* measurement of patient-specific biodistributions; these measurements could facilitate future development of novel dosimetry paradigms wherein mAb dose is titrated to optimize outcomes for individual patients.

**Methods:**

The prostate stem cell antigen (PSCA) is broadly expressed on the surface of prostate cancer (PCa) cells. Anti-human PSCA monoclonal antibodies (mAb 7F5) were bound to Au/Fe_3_O_4_ (GoldMag) nanoparticles (mAb 7F5@GoldMag) to serve as PSCA-specific theragnostic MRI probe permitting visualization of mAb biodistribution *in vivo*. First, the antibody immobilization efficiency of the GoldMag particles and the efficacy for PSCA-specific binding was assessed. Next, PC-3 (prostate cancer with PSCA over-expression) and SMMC-7721 (hepatoma cells without PSCA expression) tumor-bearing mice were injected with mAb 7F5@GoldMag for MRI. MRI probe biodistributions were assessed at increasing time intervals post-infusion; therapy response was evaluated with serial tumor volume measurements.

**Results:**

Targeted binding of the mAb 7F5@GoldMag probes to PC-3 cells was verified using optical images and MRI; selective binding was not observed for SMMC-7721 tumors. The immunotherapeutic efficacy of the mAb 7F5@GoldMag in PC-3 tumor-bearing mice was verified with significant inhibition of tumor growth compared to untreated control animals.

**Conclusion:**

Our promising results suggest the feasibility of using mAb 7F5@GoldMag probes as a novel paradigm for the detection and immunotherapeutic treatment of PCa. We optimistically anticipate that the approaches have the potential to be translated into the clinical settings.

## Introduction

Prostate cancer (PCa) is the most common cancer among men in the United States and is the second leading cause of death from cancer in men [1]. Localized PCa can be treated with surgery or radiation therapy, but the disease recurs in approximately 20 to 30% of patients. Androgen-deprivation therapy, the most common treatment after recurrence, is effective, but the disease eventually progresses in most patients who receive such treatment [2], [3], [4]. For men with metastatic PCa, median survival in recent phase 3 studies ranged from 12.2 to 21.7 months [1], [2], [3], [4]. A chemotherapeutic agent, docetaxel, is the only approved therapy shown to prolong survival among men with this condition, conferring a median survival benefit of 2 to 3 months [5], [6]. Conventional anticancer therapies, such as chemotherapy and radiation therapy, are characterized by a lack of tumor cell specificity.

Convincing evidence indicates that tumor antigen (TA)–targeted monoclonal antibody (mAb)–based immunotherapy is clinically effective for the treatment of a broad range of cancer etiologies [7], [8]. However, TA-targeted mAb-based immunotherapy has demonstrated variable clinical efficacy for the treatment of patients with PCa; this form of therapy has been effective in only a subset of the disease expressing the specifically targeted TA [9], [10]. An obstacle that is currently impeding translational progress has been the inability to quantify the patient-specific dose of mAbs that bind to the targeted TAs. This information remains largely unknown in clinical settings but would permit patient-specific dose adjustments or adoption of alternate treatment strategies when necessary (i.e. target a different antigen and/or utilize alternative mAbs).

In clinical settings, 18-F fluorodeoxyglucose (FDG) positron emission tomography (PET) provides an early and accurate way to determine if cancer is responding to treatment. Some new molecular imaging technologies with PET hold promise for PCa management. Fluorestradiol (FES) measures estrogen receptors to track tumors and fluorothymidine (FLT) provides insight into cellular growth and proliferation [11], [12]. More recently, there are other metabolic PET tracers have been successfully tested for prostate cancer [13], [14]. Importantly, there has been a study that used of humanized anti-PSCA (prostate stem cell antigen) intact antibody as a molecular imaging probe for PET imaging, which is currently under development for evaluation in a pilot clinical imaging study [13]. However, PET may provide insufficient spatial resolution for the detection of early stage of PCa [15]. The advantages of MRI over nuclear-based molecular imaging techniques include higher spatial resolution, superior soft tissue contrast, and the ability to integrate molecular, anatomic, and physiologic imaging data, all without exposing a patient to potentially harmful radionuclides. In addition, MRI provides insight into tumor function and has the potential to bridge further the divide between molecular biology and clinical translation.

Recent pre-clinical research efforts to develop multi-modality molecular imaging methods have the potential for noninvasive PCa diagnosis and imaging-guided immunotherapy [16], [17]. Multiple groups are actively pursuing the development of imaging probes for cellular and molecular MRI [12], [16], [18], [19], [20]. Superparamagnetic iron oxide (SPIO) nanoparticles can be readily bound to various molecular markers including ligands, antibodies, and peptides as MRI probes [21], [22], [23], [24]. The static magnetic field is considerably disturbed by these SPIO-based probes, the dephasing of the processing spins leads to localized signal loss in MR images.

Au/Fe_3_O_4_ nanoparticles with a shell/core structure are synthesized by reduction of Au^3+^ with hydroxylamine in the presence of Fe_3_O_4_ [25], [26]. Au/Fe_3_O_4_ nanoparticles were used for this study with an average size 50 nm (shell/core, 5/45 nm) in diameter (GoldMag Biotechnology Co., Ltd, Xi’an). Magnetic nanoparticles (Fe_3_O_4_) have attracted broad attention due to their potential applications in MRI [21], [22], [23], [24], [27]. The formation of gold shell on the magnetic nanoparticle was performed by an iterative reduction method using hydroxylamine [28], [29]. The Fe_3_O_4_ core provides a particle of a small size with significant magnetic moment, and a gold coating on the Fe_3_O_4_ core can introduce a good platform for further conjugation with biomolecules especially for biosensors fabrication. The gold-coated Fe_3_O_4_ nanoparticles were reported to exhibit good biocompatibility and affinity via amine/thiol terminal groups [28], [29]. With relative larger antibody immobilization capacity, Au/Fe_3_O_4_ nanoparticles are a good antibody carrier as compared with Au nanoparticles and Fe_3_O_4_ nanoparticles. Because of inherent high saturation magnetization, Au/Fe_3_O_4_ nanoparticles could response quickly to extrinsic magnetic field with less time consumption during separation process. Therefore, the gold-coated magnetic nanoparticles satisfy the basic requirements as immunology carrier. These Au/Fe_3_O_4_ nanoparticles require only a single step for antibody immobilization and provide relatively large and stable antibody binding capacities [20], [26], [30], [31], [32]. Antibody-conjugated Au/Fe_3_O_4_ nanoparticles could potentially serve as a sensitive theragnostic MRI probe permitting *in vivo* visualization of mAb biodistribution and targeted delivery to tumors. These techniques could ultimately allow clinicians to optimize individual dosage for improved outcomes during immunotherapy and/or permit rapid, timely adoption of alternate treatment strategies when needed.

The prostate stem cell antigen (PSCA) is a glycosyl phosphoinositol–anchored cell surface protein that belongs to the Thy-1/Ly-6 class of surface antigens. PSCA is an ideal candidate for the development of reagents for the detection or immunotherapy of PCa because it has increased expression specificity for PCa and has a cell surface location [33], [34], [35], [36]. mAb 7F5 is currently recommended for the detection of PSCA of human origin during Western Blotting, immunofluorescence and flow cytometry procedures [36], [37].

In this study, mAb 7F5 was conjugated to Au/Fe_3_O_4_ nanoparticles to produce novel MRI theragnostic probe (7F5@Au/Fe3O4) for PCa. The purpose of the study was to investigate the efficacy of this theragnostic MRI probe specifically targeting PSCA on the surface of human prostate cancer cells (PC-3 cells) for detection and immunotherapy in a mouse xenograft model.

## Materials and Methods

### Cell Lines and Animal Model

This study was conducted with the approval of the Institutional Animal Care and Use Committee. All animals were housed and handled according to the Institutional Animal Care and Use Committee guidelines and all animal work was approved by the appropriate committee (IACUC 0000000 and 0000000A-1). The protocol was approved by the local Ethics committee (ethics committee, Fourth Military Medical University 127/2008) and all animals received humane care in compliance with “The Principles of Laboratory Animal Care” formulated by the National Society for Medical Research and the “Guide for the Care and Use of Laboratory Animals” published by the National Institutes of Health (NIH Publication No. 86–23, revised 1996).

Four to six-week-old nude mice (male Bab/c mice, weighing between 25 and 30 g) were used for these studies. A human prostate carcinoma cell line; the PC-3 was initiated from a bone metastasis of a grade IV prostatic adenocarcinoma [33], [34], [35], [36]. The cells were obtained commercially from American Type Culture Collection (ATCC; Rockville, MD) and grown as a monolayer in Eagle’s minimum essential medium (Invitrogen Corp., Grand Island, New York) supplemented with 15% fetal bovine serum (FBS) at 37°C under a mixture of 95% air and 5% CO_2_. For creation of PC-3 tumor-bearing model, mouse was inoculated with 2×10^6^ cells/5ml PBS in right flank. Another tumor-bearing mouse (SMMC-7721 tumors without PSCA expression) was created to serve as control group. SMMC-7721, a human hepatoma carcinoma (HCC) cell line, was cultured in Dulbecco’s modified Eagle medium (DMEM, Invitrogen Corp., Grand Island, New York) supplemented with 10% FBS at 37°C under a mixture of 95% air and 5% CO_2_ [38].

### Construction and Evaluation of mAb 7F5@Au/Fe3O4 Theragnostic MRI Probe

MRI theragnostic probes were constructed using either PCa specific mAb 7F5 (Santa Cruz Biotechnology Inc., Santa Cruz, California) or a non-specific antibody, mouse anti-human IgG (Wuhan Boster Biology Co. Wuhan) to serve as a control probe. Both 7F5@Au/Fe3O4 and IgG@Au/Fe3O4 probes were constructed as previously described [20], [26], [30], [31], [32]. Briefly, 200 µl (1 mg/ml) of the Au/Fe_3_O_4_ nanoparticles were placed in a pipette tube. This tube was then placed in a magnetic separator for 2 min. 100 µg of antibody protein (either mAb 7F5 or IgG) was dissolved in 400 µl coupling buffer; the separated Au/Fe_3_O_4_ nanoparticles were then added to 350 µl of the antibody solution. The remaining antibody solution (50 µl) was used for coupling efficiency tests. The antibody solution with Au/Fe_3_O_4_ nanoparticles was placed in a constant temperature (37°C) shaker for 20 min at 180 rpm, moved to a centrifuge tube. This tube was placed in a magnetic separator to remove the supernatant of immobilized antibody-Au/Fe_3_O_4_ nanoparticles. 50 µl of this supernatant was used for coupling efficiency tests. The binding capacity of the Au/Fe_3_O_4_ surface was determined using a UV−vis spectrophotometer (PerkinElmer, Waltham, Massachusetts) and coupling efficiency (percentage of protein uptake) calculated: 
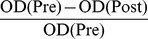
×100%, with OD(pre) and OD(post) the absorbance measurements at 280 nm for the 50 µl pre- and post-coupling antibody solutions, respectively. Specific binding was evaluated previously described [20], [26], [30], [31], [32]. In brief, separately, 5 µg/ml 7F5@Au/Fe3O4 or IgG@Au/Fe3O4 were incubated with 5×10^5^ PC-3 cells and SMMC-7721 cells for 30 min in fluorescence-activated cell sorting (FACS) buffer. The cells were washed twice with PBS, stained with fluorescein isothiocyanate (FITC) conjugated goat anti-mouse secondary antibody via incubation for 1.5 hrs (room temperature). Samples were washed twice with PBS for the evaluation of specific binding using flow cytometry assay. For optical microscopy analysis, PC-3 and SMMC-7721 cells adhered to the coverslips. Separately 200 µg/ml 7F5@Au/Fe3O4 or IgG@Au/Fe3O4 was then incubated with PC-3 cells and SMMC-7721 cells for overnight at 37°C. The cells were washed three times with PBS. The cells were stained with Cy3 conjugated goat anti-mouse secondary antibody via incubation for 1.5 hrs at room temperature. The cells were then fixed in 4% formalin for 30 min and nuclear counterstaining was accomplished with 4, 6-diamidino-2-phenylindole dihydrochloride (DAPI, Sigma, St. Louis, MO) staining in a 1.5 µg/ml solution (5 min/RT). Finally, coverslips were mounted and then visualized with laser scanning confocal microscopy (LSCM, Olympus Optical Co. Ltd., Tokyo, Japan).

### Toxicity of the 7F5@Au/Fe3O4 MRI Probe in Culturing PC-3 Cells

PC-3 cells with 93%–95% viability (trypan blue staining) were seeded in 96-well plates (4000 cells/well) with 4 ml culture medium. The following day, 20 µl mAb 7F5 or 20 µl mAb 7F5@GoldMag prob was added to PC-3 cell suspensions. In both cases, the final concentration of mAb 7F5 was the same (0.2, 2, 4, 8 and 16 µg/ml, each n = 6). The cells were treated with various mAb 7F5 concentrations (from mAb 7F5 solution or mAb 7F5@GoldMag) in 96-well plates at cell culture incubator for 24 hrs. The toxicity of equivalent amounts of GoldMag particles compared to mAb 7F5@GoldMag was evaluated as well. The final concentration of GoldMag particle was the same (2, 4, 8, 16, and 32 µg/ml, each n = 6). The cell viability (number of living cells) was measured by trypan blue staining following treatment 24 hrs. The cell inhibition rate was calculated using the formula: cell inhibition rate = (1− Npost/Npre)×100%; where Npost is the number of living cells and Npre is the number of the cells in wells pre-treated.

### Toxicity of the 7F5@Au/Fe3O4 MRI Probe in Mice

Six-week-old nude mice (male Bb/c mice, weighing between 30 and 35 g, n = 35) were used for the toxicity tests. All groups received a single dose via intravenous injection. Three groups of mice (each group n = 5) received 7F5@ Au/Fe_3_O_4_; three groups of mice (each group n = 5) received IgG@Au/Fe3O4 at a doses of 100, 200 and 300 µl respectively (normalized dose 1 mg Au/Fe_3_O_4_ with 50 µg antibody in each ml in following experiment). Additional control group received saline injections (300 µl, n = 5). The toxicity of 7F5@Au/Fe3O4 or IgG@Au/Fe3O4 probes was assessed with multiple indices. For acute toxicity, animals were observed for events such as vital signs, mental, diet and activity level following administration of the probe for 96 hrs. Systemic toxicity was evaluated using the changes in animal body weights. The weight and physical status of all the mice were monitored for a period of 30 days. The animals were weighed on the day of probe injection and every 5 days thereafter until 30 days post-injection.

### Magnetic Resonance Imaging

These studies were performed using a clinical 3.0T whole-body MR-system (Siemens Magnetom Trio, Erlangen, Germany). The system was capable of operating at a maximum slew rate of 200 mT/m/ms and a maximum gradient strength of 40 mT/m. Integrated system body coils were used for RF excitation and an eight-channel clinical head coil was used for signal reception.

### In vitro MRI of 7F5@Au/Fe3O4 Targeted Cells

PC-3 cells and SMMC-7721 cells were cultured with 7F5@Au/Fe3O4 and IgG@Au/Fe3O4 separately for 12 hrs. The concentrations of 7F5@Au/Fe3O4 and IgG@Au/Fe3O4 were 1 mg Au/Fe_3_O_4_ per 50 µg antibody for each 1 ml of culture medium with 0.5 million cells. After 12 hrs, harvested cells were washed 3 times with PBS. Cell samples were mixed with 1.5 ml 1% agarose in small centrifuge tubes containing I) PC-3+7F5@Au/Fe3O4; II) PC-3+ IgG@Au/Fe3O4; III) SMMC-7721+7F5@ Au/Fe_3_O_4_; or IV) SMMC-7721+ IgG@Au/Fe3O4 with each sample containing roughly 5×10^5^ labeled cells (each group, n = 8). Fast spin-echo T1-weighted (T1w) and T2-weighted (T2w) MRI measurements were performed using the following parameters: repetition time (TR)/echo time (TE) = 500/25 ms (T1w) and 4000/90 ms (T2w), field of view (FOV) = 156×156 mm^2^; slice thickness = 2 mm; matrix size = 192×192.

### In vivo MRI of 7F5@Au/Fe3O4 Targeted Tumors

The PC-3 and SMMC-7721 tumor-bearing mice were injected with either 200 µl (1 mg Au/Fe_3_O_4_ with 50 µg antibody in each ml) 7F5@Au/Fe3O4 via tail vein (PC-3 tumor-bearing mice, n = 8; SMMC-7721 tumor-bearing mice, n = 8) or 200 µl IgG@Au/Fe3O4 (each, n = 8). During MRI studies, mice were anesthetized with ketamine (80 mg/kg) via intraperitoneal injection (IP). MRI studies were performed before and 6, 12, and 24 hrs post-injection. Following localization scout scans, FSE T1w and T2w measurements were performed (T1w: TR/TE = 500/25 ms; T2w: TR/TE  = 4000/90 ms, slice thickness = 3 mm, FOV = 56.25×100 mm^2^; slice thickness/slab thickness = 2/12.8 mm; matrix size = 72×128). After MRI, mice were euthanized via CO_2_.

### Assessment of 7F5@Au/Fe3O4 Probe Biodistribution

Tumor tissues were harvested from six mice following 7F5@Au/Fe3O4 probe infusion (3 from each tumor-type) for histological analysis. Tumor tissues were frozen in OCT medium and sectioned at 5 µm intervals. For Prussian bluing staining, these sections were incubated in a 1∶1 solution of 10% aqueous solution of potassium ferrocyanide and 20% hydrochloric acid for 30 min.

An additional 24 mice were used for quantitative analysis of 7F5@Au/Fe3O4 and IgG@Au/Fe3O4 probe distributions (4 groups, 6 mice/group having PC-3 or SMMC-7721 tumors and receiving either the 7F5@Au/Fe3O4 and IgG@Au/Fe3O4 probe infusions). Liver, spleen and tumor samples were collected from each animal at 24 hrs post-injection. These tissues were prepared for inductively coupled plasma atomic emission spectroscopy with a CCD detector. (ICP-AES, Varian, Palo Alto, CA) by digesting the cells with 25% chloric acid and then heating the solution until a solid residue formed. The carbonaceous materials were removed and the solid dissolved in 2% HNO_3_ (nitric acid). The spectrometer detection wavelength was set to 238 nm for iron and calibrated with three different standard samples (Fe selected as the trace metal element for ICP-AES assessment of probe distribution given that Fe is principle component of the Au/Fe_3_O_4_ nanoparticles).

### Anti-tumor Efficacy of Theragnostic Au/Fe_3_O_4_ MRI Probe

15 days after tumor cell implantation, 200 µl of the 7F5@Au/Fe3O4 probe (PC-3 tumor-bearing mice, n = 15; SMMC-7721 tumor-bearing mice, n = 15) or 200 µl of the control IgG@Au/Fe3O4 probe (PC-3 tumor-bearing mice, n = 15; SMMC-7721 tumor-bearing mice, n = 15) was injected via tail vein at days 0, 4, and 8. Vital signs, mental status, diet and activity levels for each animal were observed daily Tumor size was measured in three dimensions (length, width, and height) with a caliper, and tumor volume was calculated using the tumor volume formula  = 4/3π×(length/2)×(width/2)×(height/2)[39]. The tumor volume was measured at multiple time points following administration of the probe (20, 25, 30, 35, 40, 45, 50, 55, 60 and 65 days after tumor cell implantation, probe infusion on day 15). All mice were euthanized at day 65.

### Image Analysis and Statistical Methods

Image analyses were performed using ImageJ (version 1.34s, National Institutes of Health, MD, USA). Region of interest (ROI) were drawn encompassing cross-sections of respective vials or mouse tumors (ROI included roughly 30 voxels for each phantom vial, 30 voxels for each *in vitro* cell sample tube or 40 voxels for each tumor) to measure mean T2w signal intensity (S_mean_). Separate ROI were drawn outside of the tube or within a region void of tissue (drawn at consistent positions between repeated measurements) to estimate relative noise levels based upon the standard deviation of the background signal (NSD). These calculations were used to estimate the relative signal-to-noise ratio (SNR)  =  S_mean_/NSD for each measurement. For tumor-bearing mice, these measurements were repeated for each MRI scan post-injection; these measurements were also repeated for each *in vitro* Au/Fe_3_O_4_ cell sample vial.

All statistical calculations were performed using the SPSS software package (SPSS, Chicago, IL, USA). For systemic toxicity studies, to account for variability in baseline body weights, the time course of body weight measurements for each animal were reported as a percentage of the original baseline body weight. One-way ANOVA was used to compare these adjusted body weight indices across treatment groups at each time interval post-infusion (Tukey post-hoc correction, p<0.05 considered statistically significant). Next, one-way ANOVA was used to compare a) T2w SNR measurements from *in vitro* sample vials containing different cell lines and targeting moieties and b) *in vivo* T2w SNR measurements in tumors at pre-infusion and three post-infusion time intervals (separate comparisons for PC-3 and SMMC-7221 mouse models). Similarly, one-way ANOVA was used to compare organ-specific ICP-AES measurements of probe concentration in PC-3 and SMMC-7721 tumor-bearing mice. Finally, for therapeutic efficacy studies, at each post-infusion observation interval, a Student’s t-test was used to compare tumor volume measurements between treated and control animals (p<0.05 considered statistically significant).

## Results

### Immobilization Efficiency and Specific Binding Assessment

**Figure 1:**
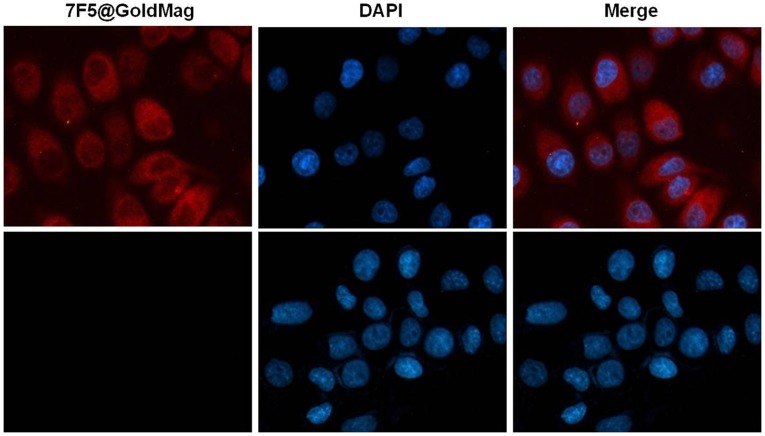
LSCM verifying specific targeting of 7F5@Au/Fe3O4 probe to PC-3 cells. Red fluorescence (7F5@Au/Fe3O4rpar; observed in the membrane of the 7F5@Au/Fe3O4+ PC-3 cells (top row) while no red fluorescence was observed in the membrane of the 7F5@Au/Fe3O4+ SMMC-7721 cells (bottom row). Cell nuclei were stained blue in color via DAPI (middle column). 7F5@Au/Fe3O4 fluorescence images and DAPI images are merged in right-most column. Scale bar, 10 µm.

The rate of antibody-immobilized coupling was gradually reduced with increasing concentrations of mAb 7F5. Adding 60 µg of mAb 7F5 to a 1 mg sample of Au/Fe_3_O_4_ nanoparticles yielded a coupling efficiency close to 83±9%; thus, for a 1 mg sample, roughly 50 µg of the mAb 7F5 was surface-coupled to the Au/Fe_3_O_4_ nanoparticles. To avoid biases during later *in vitro* and *in vivo* comparison studies, coupling efficiency was also determined for the non-specific antibody IgG. Under similar conditions, IgG to Au/Fe_3_O_4_ nanoparticle coupling efficiency was approximately 71±5% thus requiring the addition of 80 µg IgG protein to achieve a surface-coupling of 50 µg IgG to corresponding 1 mg sample of the Au/Fe_3_O_4_ nanoparticles. Flow cytometry demonstrated that the positive rate of binding was 93.6±8.2% for the 7F5@Au/Fe3O4+ PC-3 cells, while the positive rate was only 4.2±1.2% for the 7F5@Au/Fe3O4+ SMMC-7721. The positive rate of binding was 5.7±1.7% for IgG@Au/Fe3O4+ PC-3 cells and 6.9±2.1% for IgG@Au/Fe3O4+ SMMC-7721 cells. Red fluorescence was observed in the membrane and cytoplasm of the 7F5@Au/Fe3O4+ PC-3 cells with LSCM (top row in **Fig.** **1**) while no red fluorescence was observed in the 7F5@Au/Fe3O4+ SMMC-7721 cells (bottom row in **Fig. 1**). For the IgG@Au/Fe3O4 group, no red fluorescence was observed for both PC-3 cells and SMMC-7721 cell lines.

### Toxicity of the 7F5@GoldMag MRI Probe in Culturing PC-3 Cells

Toxicity of the 7F5@GoldMag MRI Probe in vitro was shown in **Fig. S1**. The toxicity of mAb 7F5 or 7F5@GoldMag was demonstrated and the cell inhibition rate increased with increasing mAb concentration; there is no statistical significance in each concentration of mAb 7F5 or 7F5@GoldMag (p>0.05 in each concentration, **Fig. S1A**). Moreover, when compared with 7F5@GoldMag group, GoldMag particle alone did not affect cell proliferation (p<0.05 in each concentration, **Fig. S1B**).

### Toxicity of Theragnostic Au/Fe_3_O_4_ Probe in Mice

**Figure 2:**
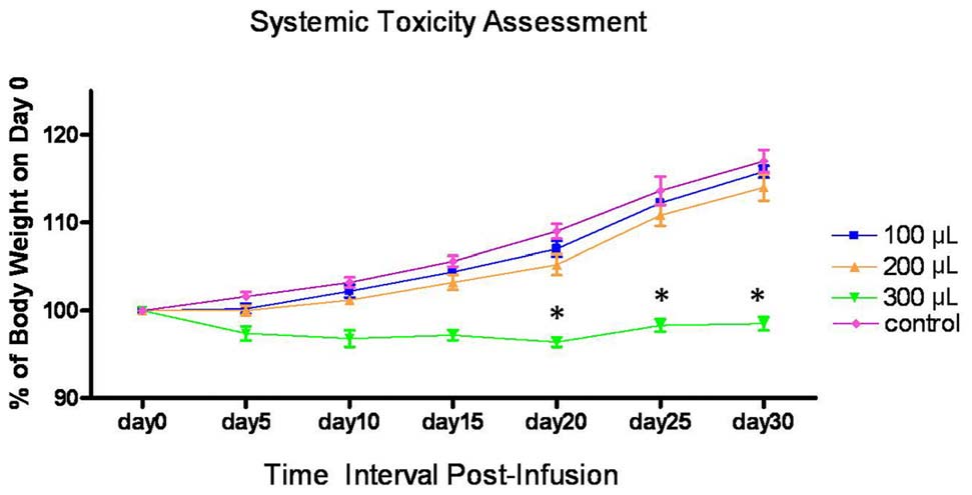
For systemic toxicity assessment, the time-coarse of animal weights were expressed as a percentage of individual baseline weight; weight changes were compared for 100 µL, 200 µL and 300 µL dose groups and a saline control group.

Acute toxicity: all mice survived and no abnormal reactions in vital signs, mental status, diet, and activity levels 96hrs after administration. Systemic toxicity in **Fig. 2**: mice in control group gained weight steadily during the course of the experiment; whereas weight gain was slightly reduced in animals receiving an infusion of 7F5@Au/Fe3O4 at doses of both 100 µL and 200 µL. No significant difference was observed between 7F5@Au/Fe3O4 and saline groups at any time point and none of these mice died during the 30-day observation period post-infusion. No clinical signs of toxicity such as trembling, decreased activity, or unstable movements were observed. Similar results were observed for IgG@Au/Fe3O4 treated animals at doses of 100 µL and 200 µL (data not shown). However, from the 20-day observation interval onward, those animals that received a 300 µL dose demonstrated significantly lower body weights compared to those animals in control and two lower dose groups (p<0.05 for each of these normalized body weight gain comparisons). One mouse in this high dose group died at day 30. Similar results were observed for the high dose IgG@Au/Fe3O4 group with two of these mice dying at days 25 and 27, respectively. Four mice from high dose groups (three mice from 7F5@Au/Fe3O4 group and one from IgG@Au/Fe3O4 group) showed clinical signs of toxicity including trembling, decreased activity and unstable movements by day 20. These results suggest increased systemic toxicity for those animals receiving a 300 µL dose of either 7F5@Au/Fe3O4 or IgG@Au/Fe3O4 probes.

### *In vitro* MRI of 7F5@Au/Fe3O4 Targeted Cells

**Figure 3:**
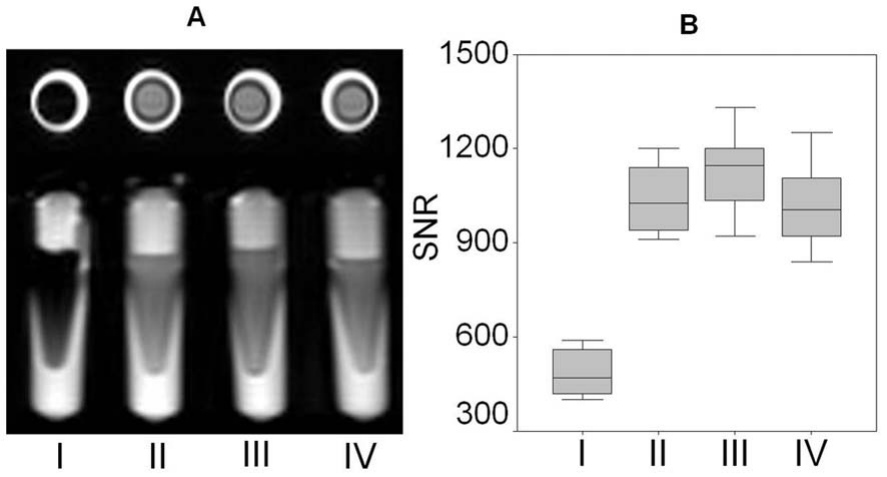
In vitro MRI of 7F5@Au/Fe3O4 targeted cells. Tube I: PC-3+7F5@Au/Fe3O4 cell sample, tube II: PC-3+ IgG@Au/Fe3O4 sample; tube III: SMMC-7721+7F5@Au/Fe3O4 sample; Tube IV: SMMC-7721+ IgG@Au/Fe3O4 sample. Quantitative T2w SNR measurements (**B**) for the cell sample vials depicted in (**A**).

*In vitro* studies demonstrated significantly greater T2w SNR reductions within PC-3+7F5@Au/Fe3O4 cell samples (tube I) compared to the PC-3+ IgG@Au/Fe3O4 cell samples, SMMC-7721+7F5@Au/Fe3O4 samples, and SMMC-7721+ IgG@Au/Fe3O4 samples within tubes II, III, and IV respectively (**Fig. 3A**). The samples within tubes II, III, and IV demonstrated no clearly appreciable SNR reductions (comparisons yielded no statistically significant differences from control, p>0.05 for each comparison); the T2w SNR within PC-3+7F5@Au/Fe3O4 cell sample (tube I) was significantly lower than the T2w SNR within sample tubes II, III, and IV in **Fig. 3B** (p<0.05 for each comparison).

### *In vivo* MRI of 7F5@Au/Fe3O4 Targeted Tumors

**Figure 4:**
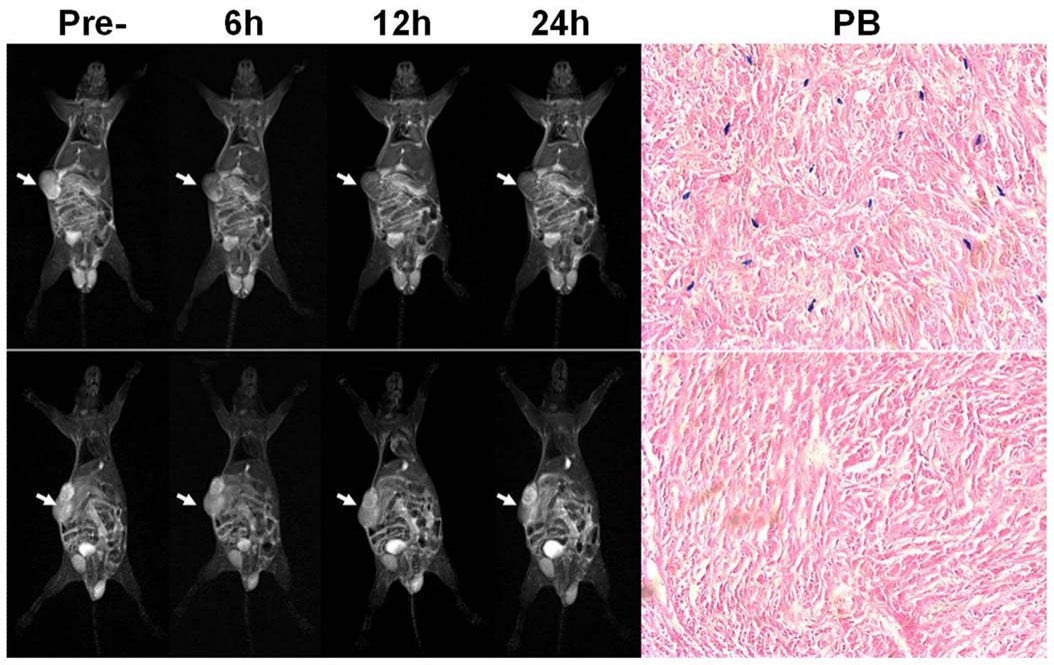
MRI of 7F5@Au/Fe3O4 targeting tumor cells *in vivo*. PC-3 tumor-bearing mice administered 7F5@Au/Fe3O4 nanoparticles demonstrated marked decreases in T2w tumor signal intensity 6, 12, and 24 hrs post-infusion (top row). No clearly appreciable tumor signal changes were observed for SMMC -7721 tumor-bearing mice at any of the three post-infusion time points (bottom row). Prussian blue staining (PB) showed that Au/Fe3O4 nanoparticles depicted as punctate blue-stained foci in the PC-3 tumor tissues; these deposits were not observed within SMMC-7721 tumor tissues. Scale bars, 10 mm for MRI and 50 µm for Prussian blue staining image.

PC-3 tumor-bearing mice administered 7F5@Au/Fe3O4 nanoparticles demonstrated marked decreases in T2w tumor signal intensity at 6, 12, and 24 hrs post-infusion (top row in **Fig. 4**). No clearly appreciable tumor signal changes were observed for SMMC -7721 tumor-bearing mice at any of the three post-infusion time points (bottom row in **Fig. 4**). Histologic analyses (Prussian blue staining in **Fig. 4**) showed heterogeneous deposition of the Au/Fe3O4 Au/Fe_3_O_4_ nanoparticles depicted as punctate blue-stained foci in the PC-3 tumor tissues; however, these deposits were not observed within SMMC-7721 tumor tissues.

**Figure 5:**
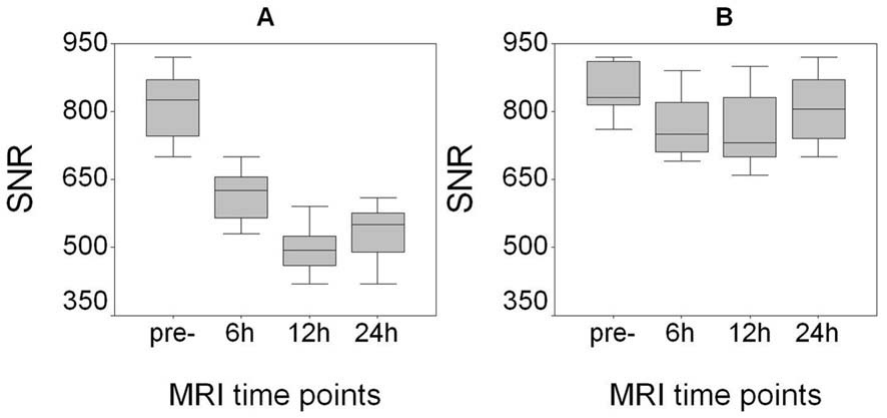
Quantitative T2w SNR measurements at 6, 12, and 24 hours post-infusion of 7F5@Au/Fe3O4 probe. T2w SNR changes (mean ± SD) in PC-3 tumors were statistically significant at each time-point post-infusion when compared to the baseline pre-infusion tumor levels (p<0.05 for each comparison) (**A**). However, there were no statistically significant changes in T2w SNR for SMMC-7721 control tumors (**B**).

Following 7F5@Au/Fe3O4 infusion, T2w SNR changes in PC-3 tumors were statistically significant when compared to the baseline pre-infusion tumor SNR levels (p<0.05 for each comparison), **Fig. 5A**. Additional significant reductions in tumor SNR were observed between the 6 and 12 hr post-infusion time points (p<0.001); while mean T2w tumor SNR later increased 24 hrs post-infusion (relative to prior measurement at 12 hrs post-infusion), this finding was not statistically significant given current sample size (p = 0.145). There were no statistically significant T2w tumor SNR changes post-injection of 7F5@Au/Fe3O4 in mice with SMMC -7721 tumors (p>0.05 for each comparison), **Fig. 5B**.

### Assessment of 7F5@Au/Fe3O4 Probe Biodistribution

**Figure 6:**
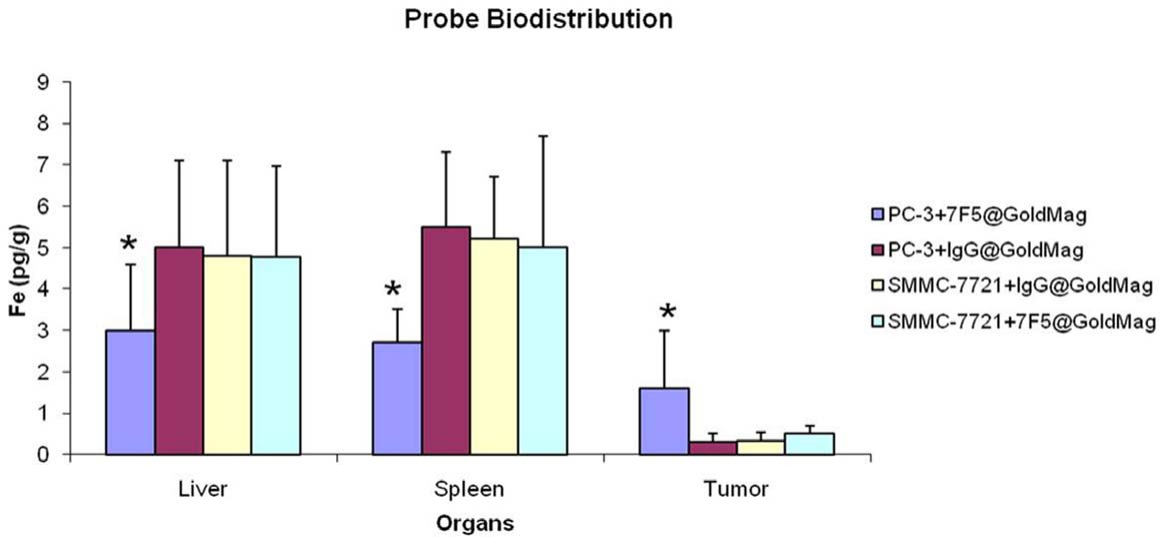
Biodistribution of the probe in vivo measurements: The probes were assessed with ICP-AES measurements of Fe concentration within liver, splenic, and tumor tissues for both PC-3 and SMMC-7721 mouse models.

**Fig. 6** shows the detected 7F5@Au/Fe3O4 and IgG@Au/Fe3O4 probe levels in liver, spleen and tumor tissues for both PC-3 tumor-bearing mice and SMMC-7721 tumor-bearing mice based upon ICP measurements of Fe content 24 hrs post-injection. These measurements reflect significant uptake of the Au/Fe_3_O_4_ probe given that endogenous Fe concentrations in murine liver and splenic tissues are orders of magnitude lower than the Fe concentrations observed for these same tissues. For both splenic and liver tissues, Fe measurements 24 hrs post-infusion were significantly higher in SMMC-7721 tumor-bearing mice and PC-3 mice following IgG@Au/Fe3O4 control probe injections compared to these same tissues in PC-3 mice following 7F5@Au/Fe3O4 injections (p<0.05 for each comparison). Conversely, Fe measurements in tumor tissues demonstrated significantly higher probe concentrations in PC-3 tumors following 7F5@Au/Fe3O4 injections compared PC-3 tumors after control probe injections and SMMC-7721 tumors following injection of either targeted or control probes.

### Immunotherapeutic Efficacy of 7F5@Au/Fe3O4 Probe

**Figure 7:**
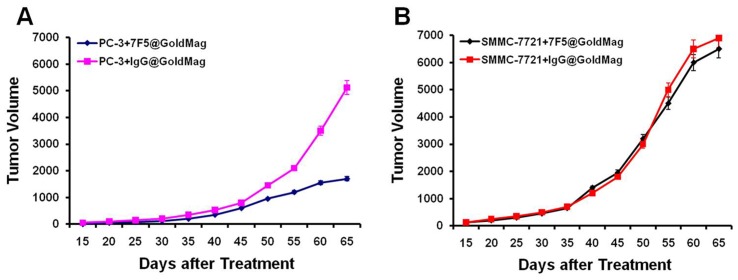
Evaluation of immunotherapeutic efficacy. For PC-3 mice, tumor volumes were significantly smaller for 7F5@Au/Fe3O4 treated animals (p<0.05) compared to those mice with IgG@Au/Fe3O4 control probe (**A**). No significant difference in tumor volume progression was observed for SMMC-7721 mice following injection of either 7F5@Au/Fe3O4 or IgG@Au/Fe3O4 probes (**B**).

Four groups of mice (7F5@Au/Fe3O4+ PC-3 tumor-bearing mice; IgG@Au/Fe3O4+ PC-3 tumor-bearing mice; 7F5@Au/Fe3O4+ SMMC-7721 tumor-bearing mice; IgG@Au/Fe3O4+ SMMC-7721 tumor-bearing mice) were administered a probe dose of 200 µl at days 0, 4, and 8. The immunotherapeutic efficacy of the 7F5@Au/Fe3O4 in PC-3 tumor-bearing mice was verified with significant inhibition of tumor growth compared to both untreated control animals and animals that received equal doses of non-targeted IgG@Au/Fe3O4 probe, **Fig. 7A**. For PC-3 mice, at 40, 45, 50, 55, 60, and 65 day observation intervals post-treatment, tumor volumes were significantly smaller for 7F5@Au/Fe3O4 treated animals (p<0.05 for volume comparisons at each of the four aforementioned intervals). No significant difference in tumor volume progression was observed for SMMC-7721 mice following injection of either 7F5@Au/Fe3O4 or IgG@Au/Fe3O4 probes, **Fig. 7B**.

## Discussion

The refinement of molecular diagnostics and the integration of these diagnostic capabilities with immunotherapy represent important steps towards personalized medicine[40]. In this study, a theragnostic MRI probe demonstrated the potential to serve a dual role for diagnosis and immunotherapy in PCa. The imaging and targeted therapy of PCa may be critical facets in effective patient management for early disease detection and selective treatment of malignant tissues. 7F5@Au/Fe3O4 theragnostic probes selectively bound to PC-3 prostate tumors in the nude mouse model. *In vivo* intravenous delivery of these probes was readily visualized with conventional T2w MRI techniques using a clinical scanner. These theragnostic probes lead to significant inhibition of PC-3 tumor growth compared to control tumors receiving equal doses of non-targeted IgG@Au/Fe3O4 probes.

The Au/Fe_3_O_4_ nanoparticles used for the current studies possess both the optical properties of gold-colloid and the superparamagnetic features of iron oxide nanoparticles [20], [30]. The latter feature produced strong T2w signal reduction proportional to Au/Fe_3_O_4_ nanoparticle concentration during our initial phantom studies. Goldmag nanoparticle with a size of 50 nm was used in our study because the core size (Fe_3_O_4_ with a size of 45 nm indiameter) is similar to clinical approved Fe_3_O_4_ particle contract agents (Feridex® or Endorem™). We have considerable experience in MRI tracking targeted cells in vivo. Moreover, GoldMag nanoparticle with a size of 50 nm was confirmed with higher antibody immobilization efficiency by review of the related GoldMag literatures [20], [25], [26], [30].

The process necessary to couple antibodies to the Au/Fe_3_O_4_ nanoparticles is relatively straight-forward requiring only commonly available laboratory equipment. Prior studies have demonstrated that the coupling of IgG and IgM to Au/Fe_3_O_4_ nanoparticles is stable and highly efficient [20], [30]. Our current findings were consistent with these prior studies.

No significant acute toxicities were observed in our mouse models within 96 hrs following administration of the theragnostic probe at dose levels of 100, 200 and 300 µl. At later time points following a 300 µl dose, systemic toxicity was observed with animals exhibiting weight loss, trembling, decreased activity, and unstable movements for both 7F5@Au/Fe3O4 and IgG@Au/Fe3O4 study groups. However, these observations were absent for both 100 and 200 µl treatment groups. The latter finding served as our rationale for using a 200 µl dose for subsequent study of the immunotherapeutic efficacy of these theragnostic probes in tumor-bearing mouse models.

PC-3 cells with over-expressing PSCA were targeted by the 7F5@Au/Fe3O4 nanoparticles for selective binding; these cells were visualized as regions of significantly reduced signal intensity within T2w MRI images. During subsequent *in vivo* studies, the 7F5@Au/Fe3O4 probe was administered intravenously to PC-3 tumor-bearing mice; at 6, 12, and 24 hrs intervals post-injection, the T2w SNR within these tumors was significantly reduced compared to pre-injection levels (greatest reduction observed at 12 hr post-injection interval). The evidence suggests the potential to use 7F5@Au/Fe3O4 nanoparticles as *in vivo* MRI probes specifically targeted to over PSCA expressing tumor cells.

ICP-AES studies to compare the biodistribution of 7F5@Au/Fe3O4 and IgG@Au/Fe3O4 probes 24 hrs post-injection revealed that the intra-tumoral uptake of the 7F5@Au/Fe3O4 probe was significantly superior (Fe concentration was 6-fold greater than that in PC-3 tumors treated with IgG@Au/Fe3O4 probe) whereas the IgG@Au/Fe3O4 control probe tended to accumulate in the liver and spleen. For non-targeted SMMC-7721 tumor model studies, both 7F5@Au/Fe3O4 and IgG@Au/Fe3O4 probes tended to accumulate in the liver and spleen as opposed to tumor tissues. These findings along with our *in vivo* time-resolved MR imaging studies suggest that the mAb 7F5 targeting moiety played a key role in eliciting selective binding to the targeted tumor cells during initial pharmacokinetic passage of the probe to reduce reticuloendothelial system (RES) sequestration within liver and splenic tissues.

Over a post-injection time-period of 50 days, significant growth inhibition was achieved for PC-3 tumors treated with a 200 µl dose, every 4 days repeated 3 times of the 7F5@Au/Fe3O4 probes. However, for SMMC-7721 tumor-bearing mice, no significant anti-tumor efficacy was observed. Similarly, no such inhibition of tumor growth was observed for tumors within either PC-3 or SMMC-7721 tumor-bearing mice after injection of IgG@Au/Fe3O4 control probe. The latter results suggest that the anti-tumor efficacy of this theragnostic probe is the result of selective binding of the mAb to the targeted PSCA antigen. Given the demonstrable impact upon tumor growth, these probes may offer important new treatment options for clinical patients with either primary or metastatic PCa.

Our promising results suggest the feasibility of using 7F5@Au/Fe3O4 probes as a novel paradigm for the detection and immunotherapy of PCa. Clearly translational studies are necessary to elucidate the sensitivity and specificity of these MRI methods for detection and/or differential diagnoses of PCa in patients. We optimistically anticipate that this theragnostic method has the potential to be translated into the clinical environment given the biocompatible gold-colloid iron-oxide composition of these nanoparticles[20], [30]. While the chosen synthesis reaction utilized for the construction of these Au/Fe_3_O_4_ nanoparticles anticipated being highly stable and reproducible; large-scale production reproducibility for manufacturing clinically relevant human dose volumes has yet to be rigorously evaluated. These factors will be critical to address prior to future translational studies intended to investigate the efficacy of these promising theragnostic PCa MRI probes in patients.

## Supporting Information

Figure S1 **Toxicity of the 7F5@Au/Fe3O4 MRI Probe in vitro.** The toxicity of mAb 7F5 or 7F5@GoldMag was observed and the cell inhibition rate increased with increasing mAb concentration; there is no statistical significance in each concentration of mAb 7F5 or 7F5@GoldMag (p>0.05 in each concentration, **Fig. S1A**). Moreover, when compared with 7F5@GoldMag group, GoldMag particle alone did not affect cell proliferation (p<0.05 in each concentration, **Fig. S1B**).
(TIF)
