# Supplementary figures and images for: MRl of Prostate Cancer Antigen Expression for Diagnosis and lmmunotherapy
Source: PLoS One. 2012 Jun 27;7(6):e38350. doi: 10.1371/journal.pone.0038350 (PMC3384648; doi:10.1371/journal.pone.0038350)

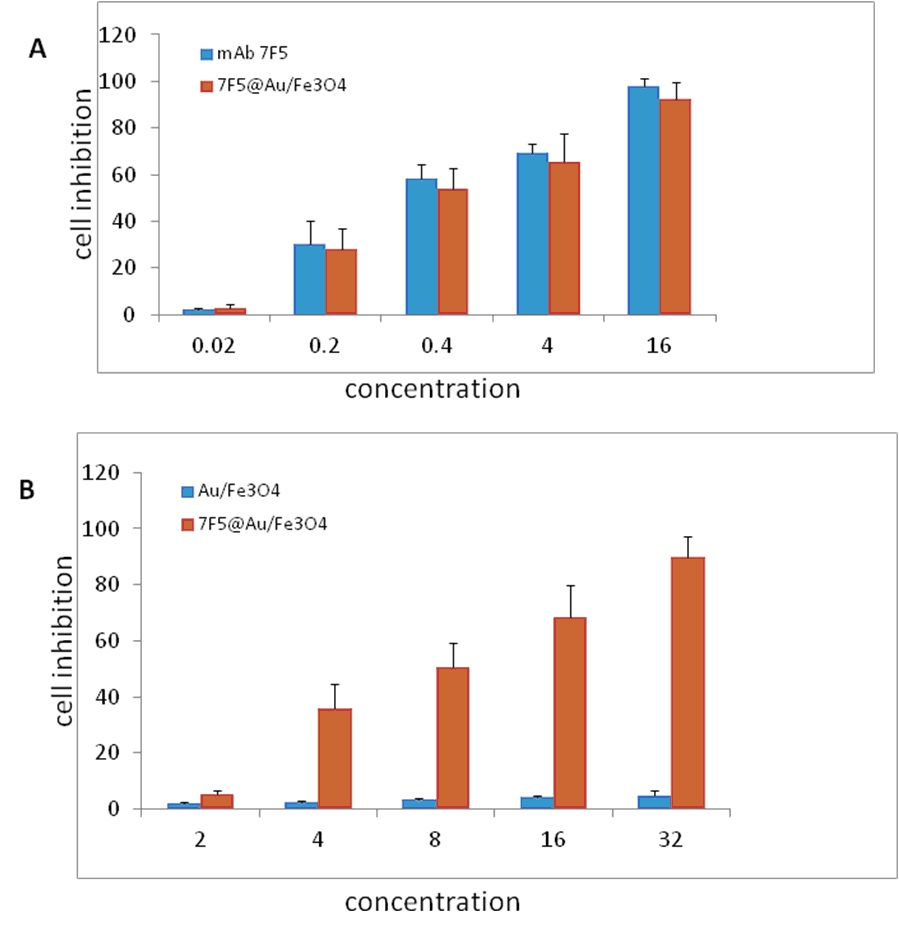

Supplement: Figure S1 — Toxicity of the 7F5@Au/Fe3O4 MRI Probe in vitro. The toxicity of mAb 7F5 or 7F5@GoldMag was observed and the cell inhibition rate increased with increasing mAb concentration; there is no statistical significance in each concentration of mAb 7F5 or 7F5@GoldMag (p>0.05 in each concentration, Fig. S1A). Moreover, when compared with 7F5@GoldMag group, GoldMag particle alone did not affect cell proliferation (p<0.05 in each concentration, Fig. S1B). (TIF) [file pone.0038350.s001.tif]
